# Clinical classification of Stargardt disease

**DOI:** 10.1007/s00417-023-06292-x

**Published:** 2023-11-07

**Authors:** Jeroen A. A. H. Pas, Patty P. A. Dhooge, Carel B. Hoyng

**Affiliations:** 1 Department of Ophthalmology, Radboud University Medical Center, Geert Grooteplein Zuid 10, 6525 GA Nijmegen, The Netherlands; 2 Donders Institute for Brain, Cognition and Behavior, Radboud University Medical Center, Nijmegen, The Netherlands

Stargardt disease (STGD1) is the most common form of juvenile macular degeneration, with a point prevalence of 1:22,000–19,000, leading to severe visual impairment or even blindness. STGD1 is caused by mutations in the ATP binding cassette subfamily A4 (*ABCA4*) gene that impair ABCA4 protein function in photoreceptor outer segments and retinal pigment epithelium cells. Over 1500 different mutations in *ABCA4* are known to cause STGD1, all with different levels of ABCA4 protein dysfunction. The high allelic heterogeneity leads to a great variety in phenotypes ranging from extensive retinal disease causing childhood blindness to late forms causing few macular abnormalities and minimal visual complaints. To date, there is no treatment for STGD1 proven to be effective. However, new therapeutic agents are currently being tested in clinical trials.

In current clinical trials, the heterogeneity in STGD1 can easily lead to conflicting outcomes. For the success of a clinical trial, the biomarker that functions as primary outcome measure must show homogeneous progression in untreated patients, to prove treatment effect during the clinical trial. To facilitate this, it is crucial to classify STGD1 so that sufficient etiologic and prognostic homogeneity is being achieved by inclusion criteria and when creating subgroups.

For centuries, scientists have been trying to classify the phenotypical spectrum of STGD1 in such homogeneous subgroups. In 1976, Gerald Fishman coined the Fishman classification, in which he described four consecutive disease stages based on fundoscopic and electroretinographic (ERG) findings. These subgroups do not adequately show disease progression as the phenotype of 66–86% of the patients remained in the same stage during a follow-up period of 4.9–7.2 years. [1] In 1998, van Driel et al. proposed a *ABCA4* genotype-phenotype correlation model [2]. When abiding to this model, patients with two severe *ABCA4* mutations are “diagnosed” with retinitis pigmentosa instead of STGD1, which can be confusing to both physicians and patients. Moreover, variable disease courses in siblings carrying the same *ABCA4* variants are frequently observed. Consequently, when using the genotype classification in a clinical trial, groups are still not homogeneous, potentially undermining the success of the clinical trial. In 2001, Lois et al. have classified STGD1 based on full-field ERG (ffERGs) [3]. Yet, STGD1 patients can present themselves without abnormalities on the ffERG and ffERGs are not sufficiently sensitive as endpoint in clinical trials for STGD1. The described disadvantages of these classifications arise from the fact that they are based on antiquated data. In recent years, huge improvements in the field of genetics were achieved, leading to more STGD1 cases, which improved the understanding of STGD1. Consequently, we introduce a new simple classification that on one hand provides both the physician and patient insight into the clinical STGD1 phenotypes and disease course, and on the other hand can be used to select the right patients and corresponding clinical endpoint for clinical trials.

In the Radboud University Medical Center (Nijmegen, the Netherlands), a cohort of around 500 STGD1 patients from 1950 till present was gathered. With the knowledge obtained by the follow-up of this large cohort, we want to propose a new classification based on the age of disease onset, i.e., the first moment a patient has symptoms related to STGD1 or in the case of incidental findings, the first moment a patient is observed with macular abnormalities that can be related to STGD1. This classification comprises three subgroups: early-onset STGD1 (age of onset ≤ 10 years old), intermediate-onset STGD1 (age of onset between 11 and 45 years old), and late-onset STGD1 (age of onset ≥ 45 years old) (Fig. 1).

**Fig. 1:**
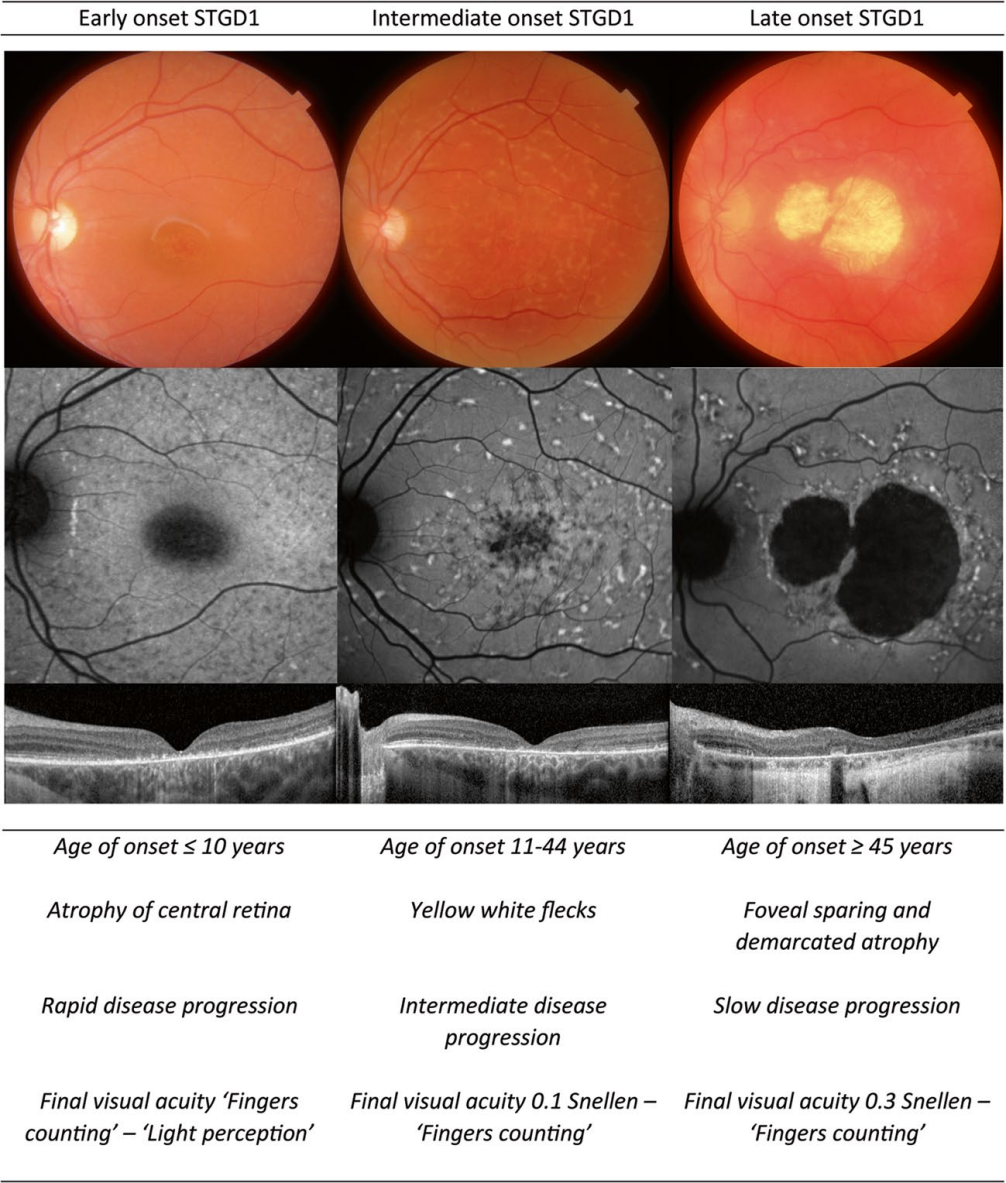
An overview of the different newly proposed subtypes of STGD1 based on the age of disease onset. This table shows their typical phenotype on fundus photography, fundus autofluorescence, and ocular coherence tomography images, and their most important features listed below the images

Early-onset STGD1 is the most severe subtype, which Lambertus et al. have already described in 2015 [4]. It is characterized by a fast disease progression accompanied with a steep drop in visual acuity in the first years of the disease. Early-onset STGD1 patients are already visually impaired by their teens. Its phenotype differs from the classical STGD1, with an absence of the typical STGD1 flecks, deep atrophy of the central retina and often a decline of visual acuity before any phenotypical changes can be noticed.

Intermediate-onset STGD1 corresponds mostly with the classical STGD1 phenotype and includes the presence of yellow-white pisciform flecks and slowly evolving central retinal atrophy. Patients often experience a steep decline in visual acuity. However, as compared to early-onset STGD1, the decline is more gradual and starts in young adulthood.

Recently, a comprehensive overview was published on late-onset STGD1, a milder form of STGD1 that is still underdiagnosed. It has a much slower disease progression, and, due to foveal sparing, the visual acuity is often preserved until years, if not decades, after the onset of disease. This subtype of STGD1 has many phenotypical similarities with age-dependent macular degeneration, increasing the chance of misdiagnosis [5].

Creating subgroups with equal disease progression rate is of vital importance for selecting the most efficient biomarkers as endpoints for upcoming clinical trials. We hypothesize that for the different subgroups, different biomarkers will reflect disease progression and thereby treatment effect best. For example, choosing visual acuity in a clinical trial with mostly late-onset STGD1 patient would be unjustifiable since the visual acuity can be preserved for years in these patients, and thereby does not show treatment effect within an acceptable clinical trial duration. Note that for early-onset STGD1, visual acuity decline seems to be most sensitive as primary endpoint while for late-onset STGD1, atrophy seems the viable option. For intermediate-onset STGD1, we hypothesize that both atrophy and visual acuity may not be the most sensitive primary endpoint and therefore suggest to use multi-modal endpoints in clinical trials (Fig. 2).

**Fig. 2:**
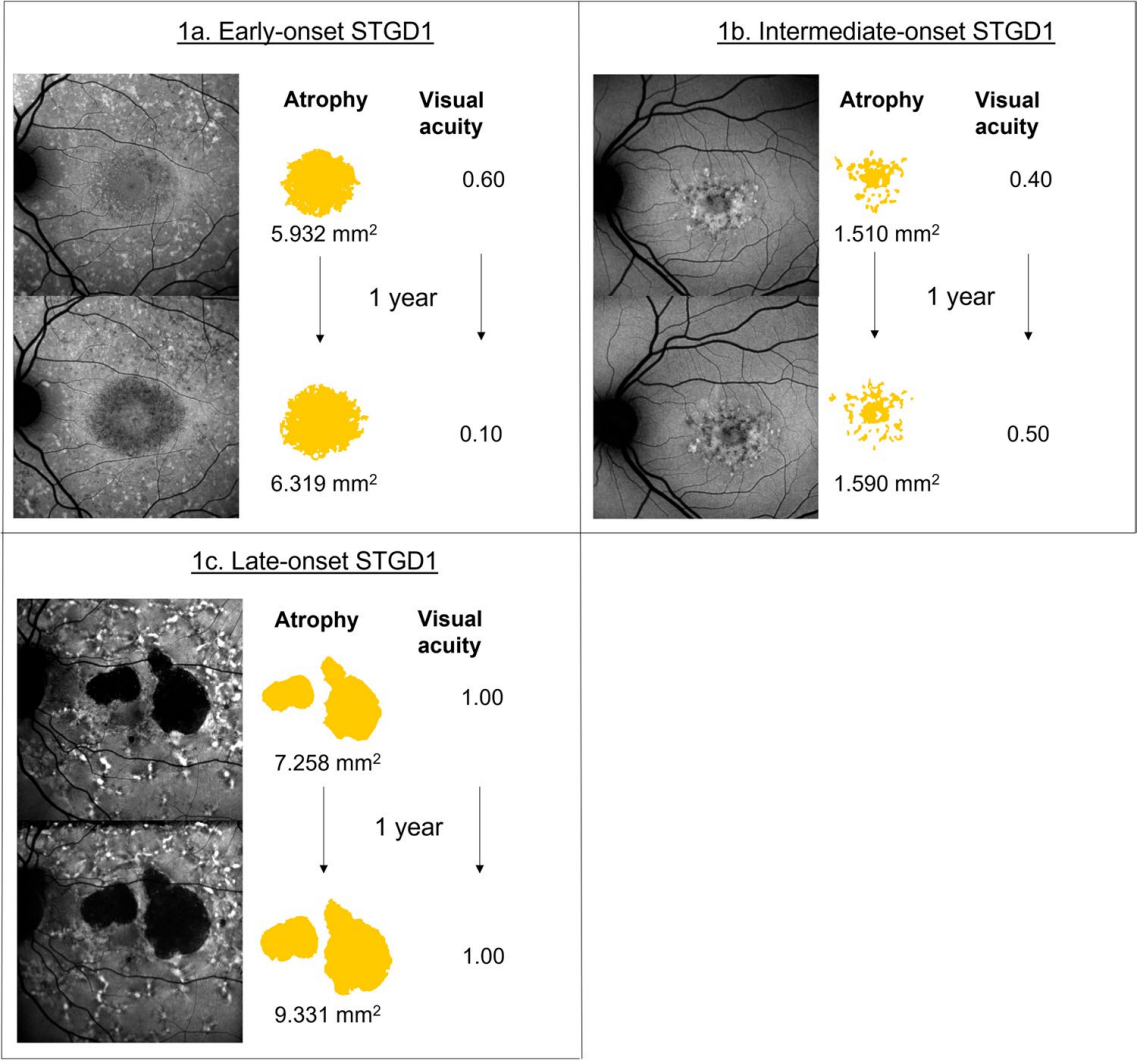
Examples of 1-year disease progression for visual acuity and atrophy size on fundus autofluorescence in the different subtypes of STGD1

We believe that our age-of onset classification provides subgroups with a more homogeneous progression rate as compared to the subgroups in previous classifications. Thereby, we hope to prevent the unnecessary failure of clinical trials due to an incorrect chosen primary endpoint, making this new classification based on age of disease onset of great value.

## Abbreviations

*ABCA4*: ATP binding cassette subfamily A4
(ff)ERG: (Full-field) electroretinogram
STGD1: Stargardt disease

## References

[1] Kim LS, Fishman GA (2006). Comparison of visual acuity loss in patients with different stages of Stargardt's disease. Ophthalmology.

[2] van Driel MA, Maugeri A, Klevering BJ, Hoyng CB, Cremers FPM (1998). ABCR unites what ophthalmologists divide(s). Ophthalmic Genetics.

[3] Lois N, Holder GE, Bunce C, Fitzke FW, Bird AC (2001) Phenotypic subtypes of stargardt macular dystrophy. Arch Ophthalmol:359–369 10.1001/archopht.119.3.359 11231769

[4] Lambertus S, van Huet RA, Bax NM, Hoefsloot LH, Cremers FP, Boon CJ, Klevering BJ, Hoyng CB (2015). Early-onset stargardt disease: phenotypic and genotypic characteristics. Ophthalmology.

[5] Li CHZ, Pas J, Corradi Z, Hitti-Malin RJ, Hoogstede A, Runhart EH, Dhooge PPA, Collin RWJ, Cremers FPM, Hoyng CB (2023) Study of late-onset Stargardt type 1 disease: characteristics, genetics and progression. Ophthalmology. 10.1016/j.ophtha.2023.08.011 10.1016/j.ophtha.2023.08.011 37598860

